# A genetically encoded multifunctional unnatural amino acid for versatile protein manipulations in living cells†
†Electronic supplementary information (ESI) available: Full experimental details and characterization data of all the new compounds. See DOI: 10.1039/c6sc02615j


**DOI:** 10.1039/c6sc02615j

**Published:** 2016-08-01

**Authors:** Yun Ge, Xinyuan Fan, Peng R. Chen

**Affiliations:** a Beijing National Laboratory for Molecular Sciences , Synthetic and Functional Biomolecules Center , Department of Chemical Biology , College of Chemistry and Molecular Engineering , Peking University , Beijing 100871 , China . Email: pengchen@pku.edu.cn; b Peking-Tsinghua Center for Life Sciences , Peking University , Beijing 100871 , China

## Abstract

The genetic code expansion strategy allowed incorporation of unnatural amino acids (UAAs) bearing diverse functional groups into proteins, providing a powerful toolkit for protein manipulation in living cells. We report a multifunctional UAA, *N* ^ε^-*p*-azidobenzyloxycarbonyl lysine (PABK), that possesses a panel of unique properties capable of fulfilling various protein manipulation purposes. In addition to being used as a bioorthogonal ligation handle, an infrared probe and a photo-affinity reagent, PABK was shown to be chemically decaged by *trans*-cyclooctenols *via* a strain-promoted 1,3-dipolar cycloaddition, which provides a new bioorthogonal cleavage strategy for intracellular protein activation. The biocompatibility and efficiency of this method were demonstrated by decaging of a PABK-caged firefly luciferase under living conditions. We further extended this method to chemically rescue a bacterial toxin OspF inside mammalian host cells.

## Abstract 2

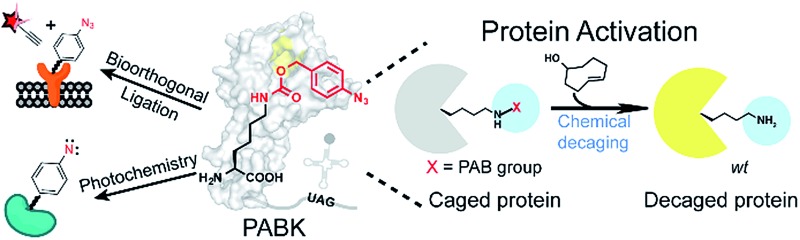
A multifunctional UAA, PABK, is developed for diverse protein manipulation purposes, especially protein activation in living cells.

## Introduction

Encoding unnatural amino acids (UAAs) bearing different functional groups, *via* genetic code expansion, offered the unprecedented capability for site-specific protein manipulation in living systems.^1,2^ The introduction of chemical functionalities on a protein of interest (POI) beyond the side chains of canonical amino acids has allowed an array of exciting applications, ranging from photo-affinity moieties for capturing intracellular protein–protein interactions (PPIs),^3,4^ and bioorthogonal handles for protein labeling and imaging in live cells,^5,6^ to various caging groups for photo or chemical triggered protein activation.^7–9^ Along with this fast expanding UAA toolkit, lowering the technical barrier for utilizing the genetic code expansion strategy would promote its accessibility to the general scientific community. For example, rather than the earlier generations of tyrosine- and leucine-based systems that are restricted to only prokaryotic or eukaryotic systems, the pyrrolysyl-tRNA synthetase (PylRS)/tRNA_CUA_ pair that has emerged and been rapidly developed in recent years allows UAA incorporation in bacteria, yeast, and mammalian cells, as well as multicellular organisms. Therefore, this Pyl-based system has become a “one-stop shop” for genetic code expansion in a diverse range of living species.^10^ Similarly, instead of many traditional UAAs that can only be used for a single purpose, we envision that a multifunctional UAA can be rationally designed to fulfill multiple purposes with the same set of chemical moieties and aminoacyl tRNA synthetase (aaRS)-tRNA pair, thus avoiding the complicated processes for synthesis, evolution or incorporation of different UAAs. Such a multifunctional UAA would offer a versatile platform for diverse downstream applications.

**Fig. 1:**
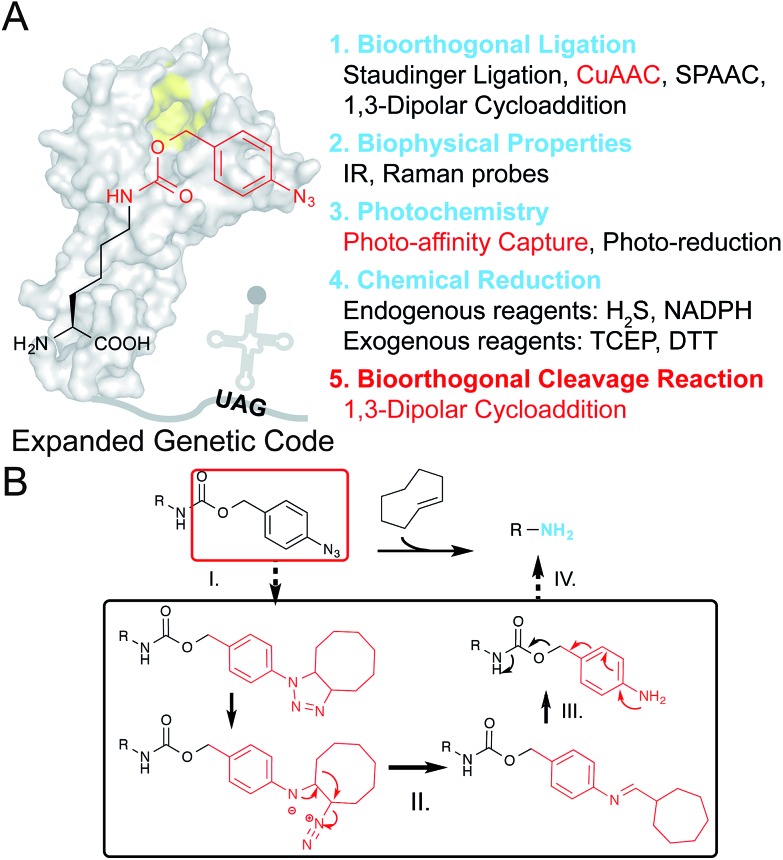
Design of PABK as a multifunctional UAA for versatile protein manipulations. (A) An evolved PylRS mutant can recognize and site-specifically incorporate PABK into the growing polypeptide chain with its cognitive tRNA in response to an amber codon (UAG). The *p*-azidobenzyl group on PABK possesses a panel of unique properties listed on the right (1–4), while coupling with a carbamate linker further renders it susceptible to the bioorthogonal cleavage reaction (5). The background shape of PylRS is drawn according to its crystal structure (PDB 2ZIN). (B) The mechanism of the protein decaging strategy based on the TCO and *p*-azidobenzyl pair: (I) strain-promoted 1,3-dipolar cycloaddition; (II) rearrangement and N_2_ release; (III) hydrolysis and (IV) 1,6-elimination. The caged primary amine can be restored by the TCO activator.

The azide moiety is one of the most widely used bioorthogonal handles that also possesses versatile uses. It can participate in a series of bioorthogonal ligation reactions for tagging biomolecules, including Staudinger ligation, copper-catalyzed azide–alkyne cycloaddition (CuAAC), strain-promoted azide–alkyne cycloaddition (SPAAC) as well as 1,3-dipolar cycloaddition.^11–15^ Meanwhile, the distinct spectral characteristics of azides make them excellent infrared (IR) and Raman probes to monitor structural or environmental information on biomolecules (Fig. 1A and Scheme S1†).^16,17^ It is noteworthy that, in comparison with aliphatic azides, the aryl-azide group can undergo unique photochemistry.^18^For example, upon UV-irradiation, N_2_ is lost from an aryl-azide to form a reactive nitrene species that will readily form a covalent bond with nearby biomolecules, generating photo-captured protein–protein interaction complexes.^19,20^ In addition, the reduction chemistry of aryl-azides, triggered by Staudinger reduction, photo-reduction^18^ or other reducing agents, converts the azide to an amine group. This method has been applied to prodrug or biomolecule activation.^21–24^Together, these properties enable the aryl-azide to serve as a multifunctional handle that can be chemically controlled to fulfill different applications.

Most recently, the strain-promoted 1,3-dipolar cycloaddition reaction between aryl-azides and strained alkenes (*e.g. trans*-cyclooctene, TCO) has emerged as a bioorthogonal azide reduction methodology, and was adapted by Gamble and coworkers for prodrug activation in living cells.^25^ The *p*-azidobenzyl group was coupled with the carbamate self-immolative linker as a bioorthogonal caging moiety for masking amine/hydroxyl groups on prodrugs. Upon addition of TCO, the unstable triazoline cycloadduct is formed, followed by a series of rearrangements and N_2_ release to produce a *p*-aminobenzyl intermediate, which will be ultimately released through a further 1,6-elimination reaction to liberate free amine/hydroxyl groups (Fig. 1B). Since this recently emerged chemical protein activation strategy is still in its infancy, partly due to the limited choices of chemical decaging reactions, we envisioned that this decaging chemistry could also be applied to protein activation in living cells. For example, the ε-amine of a given lysine residue on a protein of interest (POI) can be masked by the *p*-azidobenzyl moiety to give the inactive form, and can be chemically decaged to regain its protein function. Interestingly, although the *N* ^ε^-*o*-azidobenzyloxycarbonyl lysine (AzZLys)^26^ has been previously developed as a clickable handle for protein labeling, the steric hindrance of the *o*-azidobenzyloxycarbonyl group not only slows down its reaction rate for cycloaddition, but also limits its capturing efficiency for photocrosslinking, rendering it unsuitable for diverse protein modulations. Herein, we developed the *N* ^ε^-*p*-azidobenzyloxycarbonyl lysine (PABK) as a multifunctional UAA that acts as a ligation handle, a photocrosslinker, and a chemically-caged lysine analogue for bioorthogonal protein decaging in living cells.

## Results and discussion

**Fig. 2:**
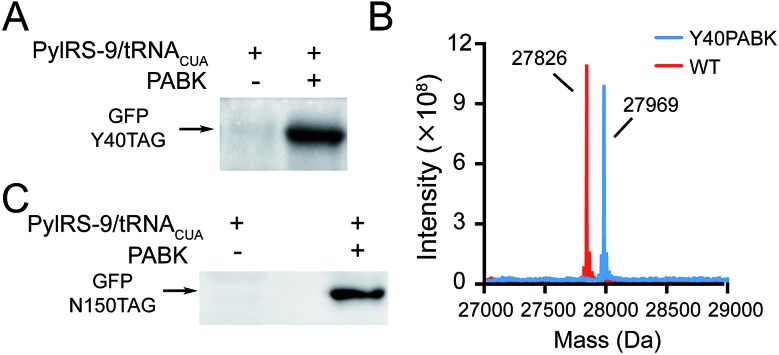
Site-specific incorporation of PABK into proteins by the Pyl-based genetic code expansion system in prokaryotic and eukaryotic systems. (A) Expression of GFP with an amber codon at Y40 and a C-terminal Flag tag in the presence and absence of 1 mM PABK with the help of PylRS-9/tRNA_CUA_ in HEK293T cells. (B) ESI-MS data for PABK incorporation into GFP at Y40 and GFP WT(Flag) as a reference. For GFP-Y40PABK(Flag): calculated 27 967 Da, found 27 969 Da; for GFP WT-Flag: calculated 27 827 Da, found 27 826 Da. (C) Expression of GFP with an amber codon at N150 and a C-terminal His tag with and without 1 mM PABK in the presence of PylRS-9/tRNA_CUA_ in *E. coli*.

We set out to synthesize PABK and surveyed PylRS variants for genetically encoding this UAA. The activated PAB intermediate was readily prepared in two steps,^21^ which then reacted with Fmoc-lysine hydrochloride salt to afford Fmoc-PABK. Deprotection of the Fmoc group gave the final product PABK (Scheme S2†). In order to encode PABK in mammalian cells, we chose to focus on four PylRS mutants that may recognize and encode PABK in the presence of its cognitive tRNA by examining the substrate binding pockets of a series of PylRS variants (Table S1†). Indeed, after testing the expression of a GFP model protein carrying an amber codon at residue Y40 in the presence of these PylRS/tRNA_CUA_ pairs, a PylRS mutant (mutations compared with wt-PylRS: L274A, C313S and Y349F, named as PylRS-9 hereafter) was found to exhibit a somewhat higher incorporation efficiency for PABK (Fig. 2A and S1†) compared to that of TCOK (TCO-caged lysine analogue)^9^ by PylRS-3 (Fig. S2†). The resulting GFP variant (GFP-Y40PABK) containing a C-terminal Flag tag was purified from HEK293T cells and subjected to ESI-MS analysis. The MS data showed an observed mass of 27 969 Da (calculated mass 27 967 Da) (Fig. 2B). With the help of PylRS-9/tRNA_CUA_, PABK was also efficiently incorporated at residue N150 in the model protein GFP in *E. coli* cells, yielding about 10 mg protein per liter LB medium (Fig. 2C). Taken together, these experiments confirmed that the PAB-caged lysine analogue PABK could be site-specifically incorporated into a POI *via* the Pyl-based system with high efficiency and fidelity.

**Fig. 3:**
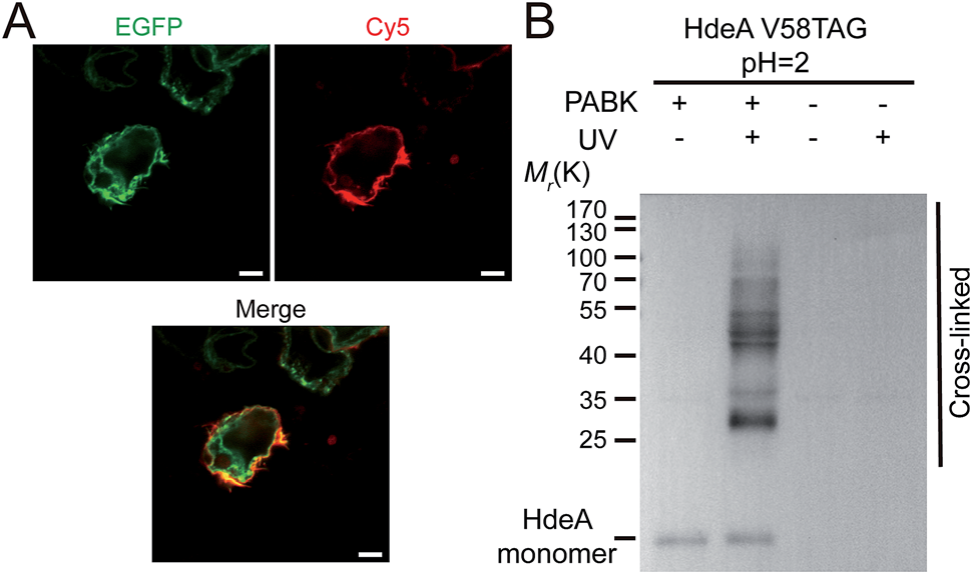
Utilization of PABK for protein bioorthogonal labeling and photocrosslinking. (A) Site-specific labeling and imaging of EGFR-N128PABK-EGFP with alkyne-Cy5 on the cell membrane by BTTAA-assisted CuAAC reaction on HEK293T cells. Top left: EGFP channel; top right: Cy5 channel; below: merged. Scale bars: 5 µm. (B) Photo-capturing HdeA's client proteins under acid stress. *E. coli* cells expressing HdeA-V58PABK-His were treated at pH 2 before being subjected to UV-irradiation. The photocrosslinked bands were shown by immunoblotting analysis using anti-His antibody.

Next we ascertained that PABK can undergo bioorthogonal ligation and photocrosslinking reactions for site-specific protein labeling and capturing protein–protein interactions, respectively. EGFR is a cell-surface receptor tyrosine kinase that is crucial in regulating cell proliferation, migration, and differentiation, as well as in tumorigenesis.^27^ Site-specific incorporation of PABK into residue Asn 128 (N128) was able to give the full-length EGFR carrying a C-terminal EGFP tag (Fig. 3A and S4†). Ligand-assisted CuAAC was used for protein labeling because the use of Cu(i) ligands such as BTTAA (2-(4-((bis((1-*tert*-butyl-1*H*-1,2,3-triazol-4-yl)methyl)amino)methyl)-1*H*-1,2,3-triazol-1-yl)acetic acid) significantly eliminated copper's toxicity while the reaction rate was further enhanced.^28^ BTTAA-assisted CuAAC reaction was performed between this EGFR variant (EGFR-N128PABK-EGFP) and an alkyne-functionalized Cy5 fluorophore (alkyne-Cy5) on HEK293T cells (Fig. 3A). The high colocalization between the EGFP channel and the Cy5 channel indicated that specific bioorthogonal labeling can be achieved on the PABK-carrying protein under living conditions. Furthermore, the fast labeling speed and the stable conjugation product formed between the purified GFP-Y40PABK protein and alkyne-Cy5 were validated by LC-MS analysis (Fig. S11†). To examine the photochemical features of PABK, we incorporated this UAA into an enteric acid chaperone HdeA that protects *E. coli* periplasm from acidic stress induced by an acidic host environment such as the human stomach (Fig. 3B and S5†). HdeA exists as a dimer at neutral pH but transforms into monomers upon acidification to turn on its chaperone activity below pH 3^4,29^ Scheme S3†). *E. coli* cells expressing the HdeA variant with PABK incorporated at residue F35 within its dimer interface (HdeA-F35PABK) were subjected to UV-irradiation at pH 7, which yielded a clearly crosslinked dimer band on western-blotting gel analysis (Fig. S12†). The efficient capturing of the HdeA dimer at neutral pH demonstrated that the phenyl-azide group on PABK undergoes photocrosslinking without decaging upon photo-activation.^30^ Moreover, *E. coli* cells expressing the HdeA variant bearing PABK at residue Val 58 in its chaperoning domain (HdeA-V58PABK) were subjected to UV-irradiation at pH 2 to initiate photocrosslinking between HdeA and its client proteins under this extreme acidity. A high photocrosslinking efficiency was observed from the immunoblotting analysis, which confirmed that PABK can be used as a valuable photo-affinity UAA for profiling PPIs in living organisms (Fig. 3B).

**Fig. 4:**
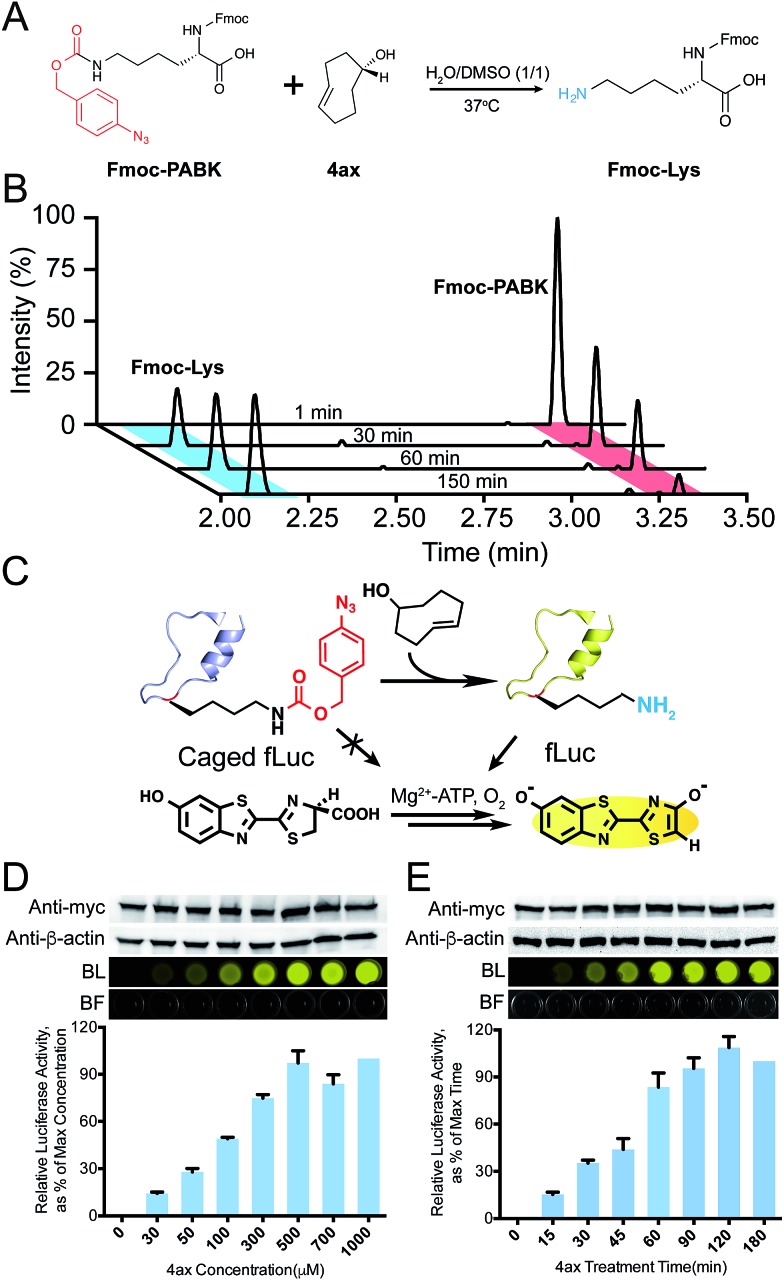
4ax-mediated bioorthogonal decaging on Fmoc-PABK and fLuc within living cells. (A) Reaction scheme between Fmoc-PABK and 4ax to generate Fmoc-lysine in H_2_O/DMSO (v/v = 1 : 1) at 37 °C. (B) Time-course study of the decaging efficiency of Fmoc-PABK's side chain in the presence of 5 equivalents 4ax as monitored by LC-MS. The peaks of both the reactant Fmoc-PABK (red) and the product Fmoc-Lys (blue) are shown. (C) Schematic representation of fLuc reactivation *via* the PABK/4ax bioorthogonal cleavage pair. The fLuc's activity which was initially caged by PABK can be rescued *via* a 4ax-mediated decaging reaction, which regenerates the wild-type fLuc activity in catalyzing luciferin oxidation with bright bioluminescence (PDB 4G36). (D) and (E) The dose- and time-dependent rescue of PfLuc activity by varying 4ax concentration at a constant incubation time (1.5 h) (D) or by varying time points with a constant 4ax concentration (500 µM) (E). fLuc was fused with a myc tag. BL: bioluminescence; BF: bright field. The histograms show bioluminescence intensity from three independent experiments. Error bars represent ±1 SEM.

Because chemical decaging of PABK is a fairly new feature in comparison with the aforementioned applications, we then focused on exploring decaging chemistry on PABK as well as its application in bioorthogonal protein activation. Gamble and coworkers have previously utilized a coumarin-based fluorogenic assay to monitor the decaging activity of three TCOs: TCO, (*S*,*E*)-cyclooct-4-en-1-ol (namely 4ax hereafter) and (*R*,*E*)-cyclooct-4-en-1-ol (4eq). They discovered that 4ax was slightly faster than 4eq in terms of the decaging rate.^25^ Since the activity of TCO is affected not only by the OH group's direction (axial or equatorial), but also by its position in the TCO ring (proximal or distal to the double bond), we decided to also evaluate the activity of TCOs with a proximal OH. To this end, we designed and synthesized two additional TCOs, (*S*,*E*)-cyclooct-2-en-1-ol (2ax) and (*R*,*E*)-cyclooct-2-en-1-ol (2eq), and compared their decaging activities with 4ax and 4eq using the same fluorogenic assay (Fig. S6†). The four TCO-OHs examined showed distinct decaging efficacies as measured by the fluorescence enhancement from the decaged coumarin (Fig. S6†). Because 4ax showed the highest elimination efficiency, it was used in the following experiments. To further validate this decaging reaction on the amino acid side chain, the decaging reaction was performed between 4ax and Fmoc-PABK (instead of PABK due to the convenience for UV detection), and was monitored by LC-MS (Fig. 4A and B). The peak corresponding to Fmoc-PABK gradually decreased in size, accompanied by an increase in the size of the peak corresponding to the decaged product Fmoc-lysine (Fig. 4B). The decaging process can be accomplished in approximately 3 h. Finally, the decaging efficiency was further evaluated on the GFP-Y40PABK protein in cell lysate which gave an approximately 90% decaged yield within 4 h (Fig. S10†).

Thus we set out to utilize the bioorthogonal cleavage pair PABK/4ax to activate an intracellular POI under living conditions. We have previously developed a firefly luciferase (fLuc)-based model system to evaluate the lysine-dependent protein activation efficiency (Fig. 4C).^31^ As expected, incorporation of PABK at residue K529 completely blocked the catalytic activity of fLuc, which also verified that the azido group on PABK remained stable in the reducing environment within the HEK293T cells. By contrast, the activity of PABK-caged fLuc (PfLuc) was effectively rescued by 4ax, yielding bright bioluminescence (Fig. S7†). Notably, the decaging reagent showed negligible influence on the activity of wild-type fLuc and exhibited no cellular toxicity at up to 1 mM final concentration for up to 72 h (Fig. S8 and S9†). Furthermore, we found that this small molecule-triggered protein activation method may be achieved in a dose- and time-dependent manner. On the basis of our initial results, we incubated cells harboring fLuc-K529PABK with 4ax at different concentrations and monitored the bioluminescence intensity at fixed times through a luciferase assay (Fig. 4D). Consistent with our hypothesis, the restored activity of PfLuc correlated well with 4ax's concentration and reached maximum signal at 500 µM after 1.5 h. Similarly, progressive activation of PfLuc over time was also observed in a time-dependent fashion (Fig. 4E). The presence of 500 µM 4ax was sufficient to restore PfLuc's activity to the maximal efficiency after 1.5 h. These dose- and time-dependent measurements established that the activity of our chemical-caged fLuc is well under the control of the PABK/4ax bioorthogonal cleavage pair. It is noteworthy that although the decaging rate of 4ax/PABK was slower than our previously reported tetrazine/TCOK decaging pair,^9^ the straightforward synthesis protocol, higher incorporation efficiency and multifunctionality make PABK an attractive “one-stop shop” for diverse applications.

**Fig. 5:**
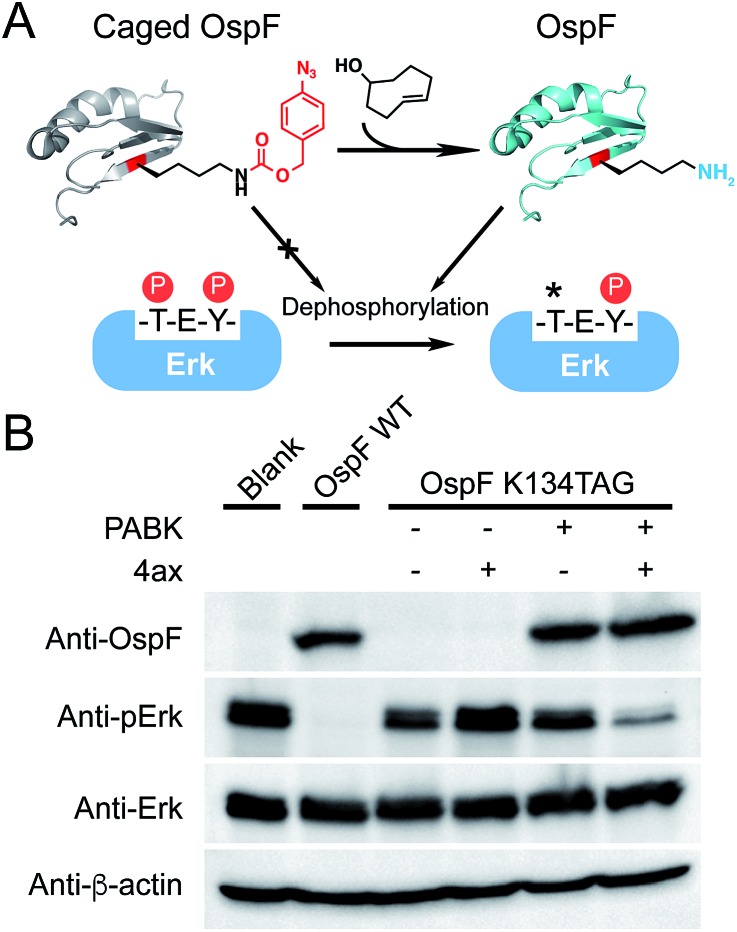
4ax-mediated activation of phosphothreonine lyase OspF in living cells. (A) Schematic representation of the manipulation of OspF's activity with PABK incorporated in place of the catalytic residue K134 (OspF-K134PABK), rendering the enzymatic activity completely masked. The addition of 4ax will effectively decage PABK, resulting in unmasked OspF activity (*e.g.* irreversible dephosphorylation of p-Erk) (PDB 3I0U). (B) The p-Erk dephosphorylation assay was performed in HEK293T cells. In contrast to wild-type OspF, cells expressing OspF-K134PABK exhibited no dephosphorylation activity on p-Erk. The addition of 4ax rescued OspF's dephosphorylation activity and leads to a decrease in p-Erk levels. 500 µM 4ax was used to treat the cells for 1.5 h.

Finally, in order to further validate the generality of this gain-of-function approach to rescue an enzyme of interest, we extended our strategy to regulate the activity of a bacterial secretion toxin. OspF is a phosphothreonine lyase secreted by enteric pathogens such as *Shigella* into host cells through a type III secretion system, which can remove the phosphate group of a mitogen-activated protein kinase (MAPK, *e.g.* Erk, p38) *via* an irreversible β-elimination reaction.^32,33^ Since OspF can specifically interfere with MAPK pathways, chemical control of its activity may provide a valuable tool to manipulate MAPK signaling pathways. Previous studies showed that Lys134 is the key catalytic residue on OspF which removes the α-hydrogen of phosphorylated Thr185 on Erk by offering a pair of electrons (Fig. 5A).^33^ PABK was site-specifically incorporated into OspF at residue K134 as an attempt to disrupt its lyase activity. As expected, the resulting OspF variant OspF-K134PABK showed a negligible influence on the phospho-Erk (p-Erk) protein. Notably, the addition of 4ax efficiently decaged PABK and the rescued OspF protein gained its dephosphorylation activity on p-Erk (Fig. 5B). In this case, specific suppression of an MAPK signaling pathway could be achieved through stimulating OspF dephosphorylation activity.^34^ This general protein activation approach has the possibility to be applied to other lysine-dependent enzymes which are significant to biological processes.

In addition, as nearly 95% of protein kinases contain a conserved lysine residue for positioning and transferring the phosphate group from ATP molecules to substrates,^36^ we decided to apply our chemical decaging method to control the catalytic activity of this crucial family of enzymes that play fundamental roles in a number of diverse cellular processes.^35–37^ For proof-of-concept, the decaging reaction was conducted for the chemical rescue of Src kinase in living HEK293T cells. The key lysine residue K295 was replaced by PABK in an oncogenic mutant form of Src (Src-Y527F) to generate the inactive Src variant (Src-K295PABK-Y527F).^38^ Upon 4ax-mediated decaging, the auto-phosphorylation of Src was clearly observed, confirming the successful rescue of oncogenic Src activity (Fig. S13†).^39,40^ This mechanism-based chemical rescue strategy may become generally applicable for kinase activation to dissect intracellular signalling transduction networks.^37^

## Conclusions

In summary, by coupling the *p*-azidobenzyl moiety with a self-immolative carbamate linker, we have developed a multifunctional UAA, PABK, as a general platform to fulfill various protein manipulation purposes. The versatile features of its functional handles enable PABK to accomplish a broad spectrum of applications, ranging from bioorthogonal protein labeling, photocrosslinking, and IR or Raman spectroscopy, to the chemical rescue of protein activity. Embarking on this genetically encoded multipurpose UAA project, we successfully performed BTTAA-assisted CuAAC labeling between a PABK-bearing EGFR and alkyne-Cy5 on a live cell surface, as well as photocrosslinking between a PABK-bearing acid chaperone HdeA and its clients within an *E. coli* periplasm. In particular, as a novel chemical-caged lysine analogue, PABK was further utilized to replace a key lysine residue on a POI, and subsequent decaging by its bioorthogonal cleavage partner 4ax ultimately led to lysine regeneration with rescue of the wild-type protein. In contrast to the more widely used loss-of-function approaches, gain-of-function methods for the functional study of POIs are exceedingly limited and highly desirable.^41,42^ Small molecule-based bioorthogonal cleavage reactions on a given amino acid side chain offer a complimentary chemical decaging approach to photo-decaging methods for gain-of-function study under living conditions.^8,9^ Our work presented here provides another appealing bioorthogonal decaging pair to mask and subsequently rescue lysine-dependent protein activity with excellent biocompatibility. This strategy can be readily applied on various lysine-dependent enzymes such as protein kinases (*e.g.* Src) and phosphothreonine lyase (*e.g.* OspF) as demonstrated in this study. Taken together, we envision that the facile synthesis scheme and the multifunctionality of this single PABK UAA, in conjunction with the Pyl-based genetic code expansion system, may become a “one-stop shop” for adding new chemical properties to proteins with diverse applications.
