# Supplementary material for: A genetically encoded multifunctional unnatural amino acid for versatile protein manipulations in living cells
Source: Chem Sci. 2016 Aug 1;7(12):7055–60. doi: 10.1039/c6sc02615j (PMC5355830; doi:10.1039/c6sc02615j)
Supplement: Supplementary file 1 [file SC-007-C6SC02615J-s001.pdf]

Electronic Supplementary Material (ESI) for Chemical Science.
This journal is © The Royal Society of Chemistry 2016

Supporting Information

A genetically encoded multifunctional unnatural amino acid for versatile protein manipulations in living cells

Yun Ge,[a]‡ Xinyuan Fan[ab]‡ and Peng R. Chen[ab]

[a] Beijing National Laboratory for Molecular Sciences, Synthetic and Functional Biomolecules Center, Department of Chemical Biology, College of Chemistry and Molecular Engineering, Peking University，Beijing 100871, China. E-mail: pengchen@pku.edu.cn

[b] Peking-Tsinghua Center for Life Sciences, Peking University, Beijing 100871, China.

‡ These authors contribute equally to this work.

Table of Contents:

Supplementary methods

1. General methods 2
2. Synthesis of PABK 2
3. Spectrofluorometry experiments 3
4. Reaction between Fmoc-PABK and 4ax monitored by LC-MS 3
5. Plasmids construction 4
6. Cell culture 4
7. Expression and purification of proteins containing PABK in *E. coli* 4
8. Expression and photocrosslinking of PABK-incorporated HdeA 5
9. Labeling of PABK-incorporated EGFR on the cell surface and fluorescence imaging 5
10. Expression and purification of PABK-incorporated GFP in HEK293T cells 5
11. ESI-MS analysis of PABK-incorporated GFP 5
12. Activation of PAB-caged fLuc in living cells 6
13. 4ax-induced OspF activation *in vivo* 6
14. Cytotoxicity study of 4ax 6
15. Quantification of labeling efficiency on PABK containing GFP 6
16. Expression and activation of PABK incorporated Src in living cells 7

Supplementary Table 8

Supplementary Schemes 8

Supplementary Figures 9

References 19

Supplementary methods

1. General methods

All chemicals were obtained from J&K Co. unless otherwise specified. NMR spectra were recorded on a Bruker 400 MHz Fourier transform spectrometer at Peking University, China. ^{1}H and $^{13}C\{^{1}H\}$ NMR spectra were referenced to residual solvent. LC-MS analysis was performed using Waters ACQUITY UPLC I-Class SQD2 MS spectrometer with electrospray ionization (ESI). Compounds **1**,[1] **2**[2] and *trans*-cyclooctenes[3] were prepared based on literature procedures. Monoclonal antibodies against myc-tag (#2276), p-Erk (#9101), Erk (#4695), pY416-Src (#6943) were purchased from Cell Signaling Technology, Inc. Antibodies against Flag (ab1162) was purchased from Abcam plc. Antibody against β-Actin (TA-09) was purchased from ZSGB-BIO. Antibody against OspF is a generous gift from Prof. Feng Shao (NIBS, China). HRP-linked secondary antibodies were purchased from CST. PMA was purchased from Promega. Alkyne-Cy5 dye was purchased from Click Chemistry Tools (Scottsdale, AZ, USA). Protein purifications were performed on an AKTA START protein purification system (GE Healthcare). Western blot images were captured on a ChemiDoc XRS+ molecular imager (Bio-Rad) or Tanon 5200 Multi image system (Tanon). Luminescence of cell samples were acquired on a Synergy H4 microplate reader (Bio-Tek). Confocal microscopy images were obtained on LSM 700 laser scanning confocal microscope (Zeiss).

2. Synthesis of PABK

2.1. (N^{2}-(((9H-fluoren-9-yl)methoxy)carbonyl)-N^{6}-(((4-azidobenzyl)oxy)carbonyl)-*L*-lysine) preparation (Fmoc-PABK)

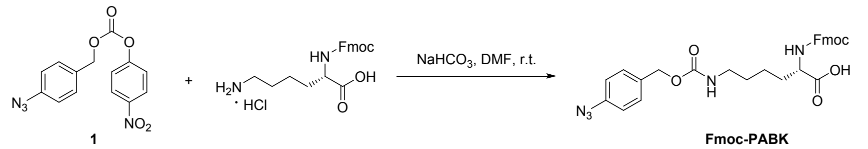

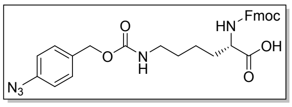

To a mixture of **1** (1.0 eq., 2 mmol, 630 mg), Fmoc-lysine (HCl) (1.2 eq., 2.4 mmol, 972 mg) and $NaHCO_3$ (3.0 eq., 6 mmol, 504 mg) in a 25 mL flask, 4 mL DMF was added. The mixture was stirred at room temperature for 8 hours. After completion, the solvent was evaporated under reduced pressure. And the crude product was purified by flash column chromatography on silica gel using DCM and MeOH as eluent, followed by second flash column chromatography on silica gel using EtOAc and hexanes as eluent. 511 mg (47% yield) of **Fmoc-PABK** was obtained as colorless oil. ^{1}H NMR (DMSO-d_6, 400 MHz) δ 7.88 (d, J = 7.5 Hz, 2H), 7.67 (d, J = 7.4 Hz, 2H), 7.41 - 7.29 (m, 6H), 7.25 (t, J = 5.4 Hz, 1H), 7.09 (d, J = 8.4 Hz, 2H), 6.50 (d, J = 6.8 Hz, 1H), 4.96 (s, 2H), 4.33 - 4.24 (m, 1H), 4.24 - 4.15 (m, 2H), 3.63 (dd, J = 11.6, 6.2 Hz, 1H), 2.93 (dd, J = 12.8, 6.5 Hz, 2H), 1.50 - 1.12 (m, 6H) ppm; $^{13}C\{^{1}H\}$ NMR (DMSO-d_6, 100 MHz) δ 174.5, 156.0, 155.2, 144.1, 140.7, 138.8, 134.3, 129.6, 127.6, 127.1, 125.2, 120.1, 119.0, 65.2, 64.5, 55.6, 46.8, 40.5, 32.4, 29.6, 22.5 ppm; HRMS *m/z* 544.21950 $(M+H)^+$; calcd for $C_{29}H_{30}N_5O_6$: 544.21906.

2.2. Preparation of (N^{6}-(((4-azidobenzyl)oxy)carbonyl)-*L*-lysine) (PABK)

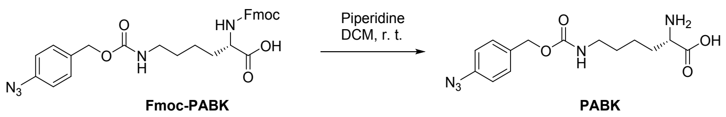

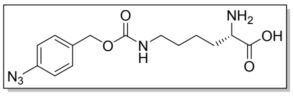

Fmoc-PABK (0.5 mmol, 260 mg) was dissolved in DCM (8 mL), piperidine (1.6 mL) was added slowly at room temperature. The mixture was stirred for 1 hour. The volatile materials were evaporated under reduced pressure, and the residue was purified by flash column chromatography on silica gel using DCM and MeOH as eluent. For using in cells, the product was purified again using reverse phase HPLC (eluent = MeCN/H_2O, gradually changing from 5/95 to 100/0 in 20 minutes). After solvent removal under reduced pressure, the residue was then dissolved in 1 mL MeOH with 10 μL concentrated HCl, and precipitated by adding Et_2O. The solid was collected and washed with MeCN (3 × 1 mL). The product was obtained as a pale yellow solid, 35 mg (20% yield). ^1H NMR (CD_3OD, 400 MHz) δ 7.38 (d, J = 8.3 Hz, 2H), 7.05 (d, J = 8.4 Hz, 2H), 5.04 (s, 2H), 3.95 (t, J = 6.2 Hz, 1H), 3.14 (t, J = 6.7 Hz, 2H), 2.05 - 1.80 (m, 2H), 1.63 - 1.35 (m, 4H) ppm; $^{13}C\{^1H\}$ NMR (CD_3OD, 100 MHz) δ 171.8, 158.9, 141.2, 135.3, 130.6, 120.0, 66.8, 53.9, 41.3, 31.2, 30.4, 23.2 ppm; HRMS *m/z* 322.15044 $(M+H)^+$; calcd for $C_{14}H_{20}N_5O_4$: 322.15098.

3. Spectrofluorometry experiments

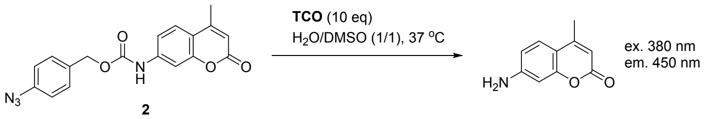

Stock solutions: A: Coumarin probe **2** (10 mM in DMSO); B: TCO-OH (10 mM in DMSO).

General procedure: 10 μL stock B was mixed with 89 μL DMSO and 100 μL H_2O. 1 μL stock A was added and the fluorescence intensity (F1) was measured by Synergy 4 Hybrid Microplate Reader at given time points (ex. 380 nm, em. 450 nm). As a control experiment, the fluorescence (F2) for the maximum amount of coumarin which could be released from probe **2** was also measured at the same time. The decaging efficiency (%) equals to F1/F2×100%. The experiments were repeated for three times.

4. Reaction between Fmoc-PABK and 4ax monitored by LC-MS

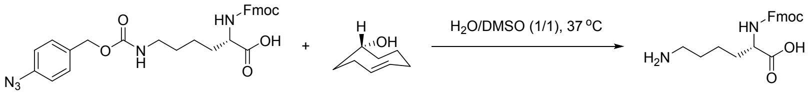

0.1 μmol Fmoc-PABK, 0.2 μmol Tyr-OMe and 0.5 μmol TCO-OH in 100 μL solvent (H_2O/DMSO = 1/1) were mixed in a vial and shaked under 37 °C. And small portion of the reaction mixture was injected into LC-MS directly (2 μL) and determined at given time point. LC-MS condition: flow rate 0.4 mL/min, eluent MeCN (0.1% AcOH)/H_2O (0.1% AcOH) gradually changing from 5/95 to 100/0 in 5 minutes, retention time (Fmoc-PABK: 3.31 min, Fmoc-K: 2.11 min, Tyr-OMe (internal standard) 1.03 min). The LC spectra are shown below.

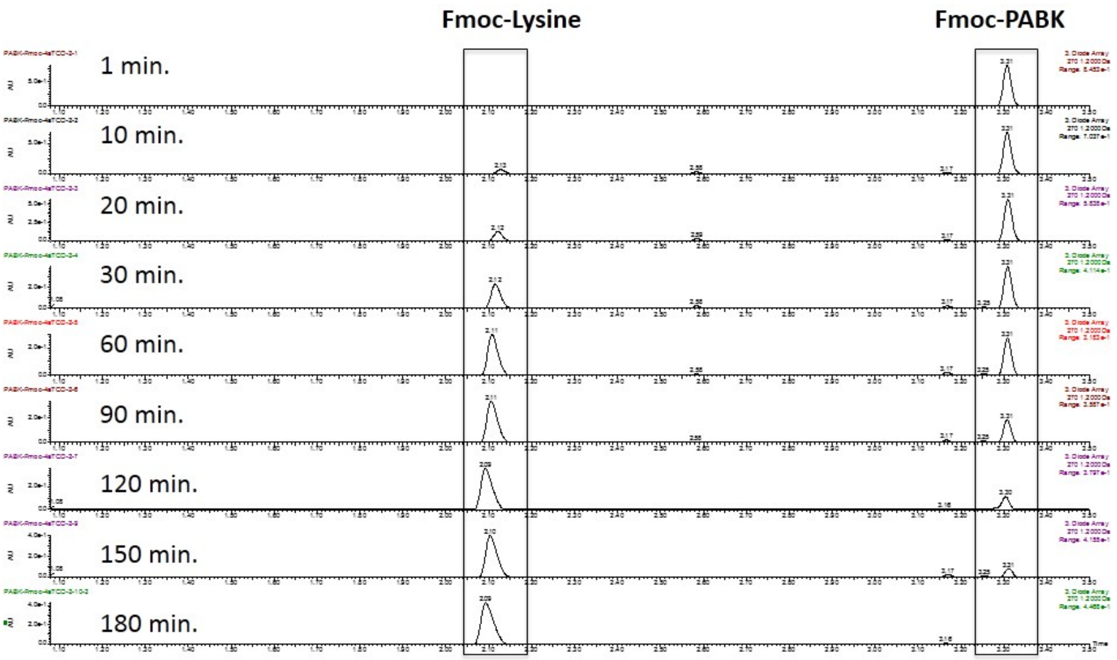

5. Plasmids construction

The pCMV-*Mb*PylRS/tRNAPyl CUA encoding mutant *Mb*-PylRS and the cognitive tRNAPyl CUA with different mutation sites (Table S1) were used in this work. pCDNA3.1-GFP-Y40TAG-His_6, pCDNA4-GFP-Y40TAG-Flag, pBAD-GFP-N150TAG-His_6 were used for experiments on model protein GFP. pCDNA4-Luc-WT-myc and pCDNA4-Luc-K529TAG-myc were utilized in luciferase assay. pcDNA4-Src-Y527F, pcDNA4-Src-K295R-Y527F, pcDNA4-Src-K295TAG-Y527F were all constructed on the pcDNA4-mycHisB vector and utilized for Src activation. pCDNA3.1-Flag-HA-OspF-K134TAG and pCDNA3.1-Flag-HA-OspF-WT are generous gifts from Prof. Feng Shao (NIBS, China). pSupAR-Mb-DiZPK-RS (the same mutations as PylRS-9), pBAD-HdeA-V58TAG-His_6, pBAD-HdeA-F35TAG-His_6 and pEGFP-N1-EGFR-N128TAG have been reported in our previous work.[4,5]

6. Cell culture

HEK293T cells were purchased from the American Type Culture Collection (ATCC). Cells were grown in DMEM (Dulbecco's modified Eagle's medium, Gibco) contained with 10% fetal bovine serum (FBS) and 100 μg/ml penicillin/streptomycin, and cultured at 37 °C under 5% CO_2.

7. Expression and purification of proteins containing PABK in *E. coli*

The plasmids pSupAR-Mb-DiZPK-RS and pBAD-GFP-N150TAG-His_6 (or pBAD-HdeA-V58TAG-His_6) were co-transfected into *E. coli* DH10B cells. Cells were recovered in 1 ml LB medium for 1 h at 37 °C before further growth in LB medium containing ampicillin (50 mg/ml) and chloramphenicol (34 mg/ml) overnight at 37 °C. After 1:100 dilutions in 100 ml LB medium containing ampicillin (50 mg/ml) and chloramphenicol (34 mg/ml), the cell culture was grown at 37 °C to an OD_{600} ~0.6, at which point 1mM PABK was added to the culture (final concentration). The bacteria were grown at 37 °C for 30 min before being transferred to 30 °C for induction in the presence of 0.02% arabinose for 12 h. The bacteria were collected by centrifugation (4500 rpm, 10 min) followed by resuspension in lysis buffer (20 mM Tris-HCl, pH 7.8, 500 mM NaCl). Lysate

after sonication was loaded onto a Ni-NTA column (Histrap 5 ml, GE Healthcare), which was washed with 30 ml washing buffer (20 mM Tris-HCl, pH 7.8, 500 mM NaCl, 40 mM imidazole) and then eluted with elution buffer (20 mM Tris-HCl, pH 7.8, 500 mM NaCl and 250 mM imidazole) to yield target protein carrying PABK. Target proteins were then desalted into DPBS.

8. Expression and photocrosslinking of PABK-incorporated HdeA

Plasmids pSupAR-Mb-DiZPK-RS and pBAD-HdeA-V58TAG-His_6 or pBAD-HdeA-F35TAG-His_6 were co-transfected into *E. coli* DH10B cells. HdeA expression was described above. 1 ml of the cultures were treated by acid-stress buffer (10 mM Sodium Citrate, 150 mM NaCl, pH = 2) for 20 min then transferred into 24-wells. For pH 7 condition, 1 ml of the cultures were resuspended in PBS buffer. Photocrosslinking was performed by irradiation of the bacterial cells with UV light (365 nm) for 10 min using a Hoefer UVC 500 Crosslinker. The cells were collected by centrifugation then analyzed by SDS-PAGE and immunoblotting.[4]

9. Labeling of PABK-incorporated EGFR on the cell surface and fluorescence imaging

Plasmids pEGFP-N1-EGFR-N128TAG and pCMV-*Mb*PylRS/tRNAPyl CUA were co-transfected via X-tremeGENE HP (Roche) in DMEM (10% FBS) supplemented with 1 mM PABK. HEK293T cells then grew for additional 24 hours to express EGFR bearing PABK. Cells were then washed three times by DPBS to remove medium and free PABK, then reacted with Alkyne-Cy5 (50 μM) in the presence of $CuSO_4$ (50 μM), BTTAA (300 μM), and sodium ascorbate (2.5 mM) in DPBS for 5 min at room temperature (RT). The reaction was quenched by the addition of BCS (500 μM). The cells were washed three times by DPBS again and fixed with 4% PFA before fluorescence imaging. Cell images were captured by an LSM 700 laser scanning confocal microscope (Zeiss) equipped with a 63× oil immersion objective lens. EGFP and Cy5 channel was chosen with corresponding parameters.[5]

10. Expression and purification of PABK-incorporated GFP in HEK293T cells

For GFP-Y40PABK expression in HEK293T cells, plasmids pCDNA4-GFP-Y40TAG-Flag and pCMV-*Mb*PylRS/tRNAPyl CUA were co-transfected via X-tremeGENE HP (Roche) in DMEM (10% FBS) supplemented with 1 mM PABK. Cells then grew for additional 24 hours to express the desired protein bearing PABK at Y40 residue. Cells were collected by centrifugation (1000×g, 5 min) at 4 °C then resuspended in PBS buffer. Lysate after sonication was centrifuged at 12000 rpm for 5 minutes at 4 °C. Supernatants were incubated with ANTI-FLAG® M2 Magnetic Beads (Sigma-Aldrich) according to its procedures for purification of GFP-Y40PABK(Flag). For analysis of decaging efficiency on GFP-Y40PABK(Flag), supernatants mentioned above were incubated with 500 μM 4ax at 37 °C for 4 h, followed by purification procedures.

11. ESI-MS analysis of PABK-incorporated GFP

LC-MS analysis of GFP-Y40PABK(Flag) was performed using a Waters ACQUITY UPLC I-Class SQD 2 MS spectrometer with electrospray ionization (ESI). 0.1% formic acid in H_2O as buffer A and 0.1% formic acid in acetonitrile as buffer B were used as the solvent system. LC separation for protein sample was carried out with a BEH300 C4 Acquity column (1.7 m, 2.1 × 100 mm), and positive mode was chosen for ESI-MS to analyze all samples. The total mass of proteins was calculated using MassLynx V4.1 software (Waters). Theoretical mass of the wild-

type protein was calculated using Peptide Mass Calculator (http://www.peptidesynthetics.co.uk/tools/), and the theoretical mass for all modified proteins was adjusted manually.

12. Activation of PAB-caged fLuc in living cells

For PABK-incorporated fLuc (PfLuc) expression in HEK293T cells seeded in a 24-well corning plate, plasmids of pCDNA4-Luc-K529TAG-myc and PylRS-9/tRNAPyl CUA were co-transfected via X-tremeGENE HP (Roche) in DMEM (10% FBS) supplemented with 1 mM PABK. Cells expressing PfLuc were treated with 4ax in different conditions to undergo the strain-promoted 1,3-dipolar cycloaddition reaction after 24 h transfection and expression. Cells were collected and lysed by 50 μL 1× Universal lysis buffer. 20 μL cell lysate were mixed with 80 μL luciferin solution (containing 20 mM Tricine, 1.0 mM $MgSO_4$, 0.1 mM EDTA, 270 μM coenzyme A, 500 μM D-luciferin and 530 μM ATP, pH 7.8) in a 96-well black plate. Relative bioluminescence unit was measured by Synergy 4 Hybrid Microplate Reader with integration time 5s and sensitivity at 120. Then bioluminescence images were taken by ChemiDoc using chemiluminescent channel. The left cell lysate was mixed with 5× loading buffer and boiled at 95 °C for 20 min. The crude sample was prepared for SDS-PAGE analysis to quantify the amount of the protein after further centrifugation.

13. 4ax-induced OspF activation *in vivo*

For OspF-K134PABK expression in living cells, pCDNA3.1-Flag-HA-OspF-K134TAG and PylRS-9/tRNAPyl CUA were co-transfected by X-tremeGENE HP (Roche) in DMEM (10% FBS) supplemented with 1 mM PABK. HEK293T cells continued to grow for another 24 h. Cells were then incubated with 4ax at 500 μM for 1.5 h. Then the culture medium was changed to fresh DMEM containing 5 μM PMA to activate p-Erk for 20 min. Cells were then collected by centrifugation (1000×g, 5 min) and lysed by lysis buffer (40 mM Tris·HCl, pH 7.5, 500 mM NaCl, 1% NP-40, 0.1% SDS, 0.5% sodium deoxycholate) containing protease inhibitor cocktail on ice for 30 min. Cell debris was discarded after centrifugation (13000 rpm, 5 min, 4 °C) and the supernatant was added with 5× loading buffer and boiled at 95 °C for 20 min. The sample was centrifuged at 13000 rpm for 10 min before being analyzed by 12% SDS-PAGE. Western blotting analysis was carried out to detect the full-length OspF protein as well as the dephosphorylation level of p-Erk by using antibodies against OspF, p-Erk1/2 and Erk1/2.

14. Cytotoxicity study of 4ax

HEK293T cells were seeded at a density of ~2,000–5,000 cells per well in flat-bottomed 96-well plates and continued to grow for 18 h. Cells were then incubated with different concentrations of 4ax for 3h and 72h. After incubation, each well's solution was replaced into the mixture of fresh DMEM and CellTiter 96 Aqueous One Solution Reagent (Promega, Madison, WI) according to the manufacturer's instructions. The cell viability was determined by measuring the absorbance at 490 nm using a microplate reader. Absorbance results at 630 nm were used to subtract background.

15. Quantification of labeling efficiency on PABK containing GFP

PABK containing GFP purified from HEK293T cells (10 μM final concentration) were labeled by Alkyne-Cy5 (100 μM final concentration) via the Cu(I)/BTTAA (BTTAA = 2-(4-((bis((1-*tert*-butyl-1*H*-1,2,3-triazol-4-yl)methyl)amino)methyl)-1*H*-1,2,3-triazol-1-yl)acetic acid) (100 μM final concentration, Cu/BTTAA = 1:6) mediated CuAAC reaction in PBS buffer. Reactions were quenched by 5 mM BCS at different time points. Then the protein was desalted and monitored by LC-MS.

16. Expression and activation of PABK incorporated Src in living cells

Plasmids of pCDNA4-Src-K295TAG-Y527F-myc and PylRS-9/tRNAPyl CUA were co-transfected via X-tremeGENE HP (Roche) in DMEM (10% FBS) supplemented with 1 mM PABK or CbzK. pCDNA4-Src-K295R-Y527F-myc, pCDNA4-Src-Y527F-myc and vector were transfected separately. After expression for 24 h, HEK293T cells were treated with 500 μM 4ax for 3 h then collected for SDS-PAGE and immunoblotting analysis. Full length of Src and its auto-phosphorylation can be detected by antibodies against myc and pY416-Src separately.

Supplementary Table

*Mb*PylRS/ *Mm*PylRS	Mutation sites			
	271/306	274/309	313/348	349/384
WT	Y	L	C	Y
PylRS-3	A	L	C	F
PylRS-4	M	A	A	F
PylRS-6	M	G	A	Y
PylRS-9	Y	A	S	F

Table S1. A list of Pyrrolysyl-tRNA synthetases (PylRS) mentioned in this work. Different mutations were shown compared with WT-PylRS.

Assay	Reaction substrates	Reaction time	Reaction efficiency*
Chemical decaging (with 4ax)	Fmoc-PABK	< 3h	100%
	PAB-coumarin	23h	53%
	fLuc-K529PABK	1.5h	Maximum signal
	GFP-Y40PABK	4h	≈90%
Click ligation (with Cy5-Alkyne)	GFP-Y40PABK	3min	>90%
		<10min	100%

Table S2. All reaction time and efficiencies shown in this work are compiled in this table. Chemical decaging and ligation have been proceeded on various substrates. *: the reaction time and efficiency listed here only in accordance with the reaction conditions described in the manuscript.

Supplementary Schemes

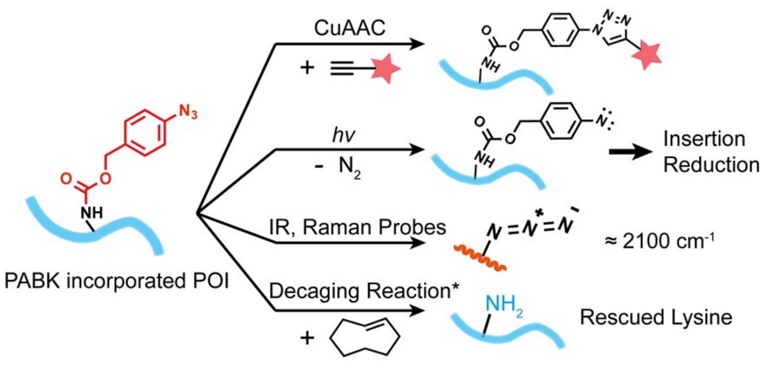

Supplementary Scheme 1. Schematic illustration of functions of PABK incorporated protein of interest (POI). PABK incorporation into POI via genetic code expansion has versatile applications. PABK can undergo different chemistries, including bioorthogonal ligation (CuAAC),

photochemistry for insertion or reduction, and decaging reaction with *trans*-cyclooctene (TCO). PABK also owns characteristic infrared band at about 2100 cm^{-1} (Fig. S16).

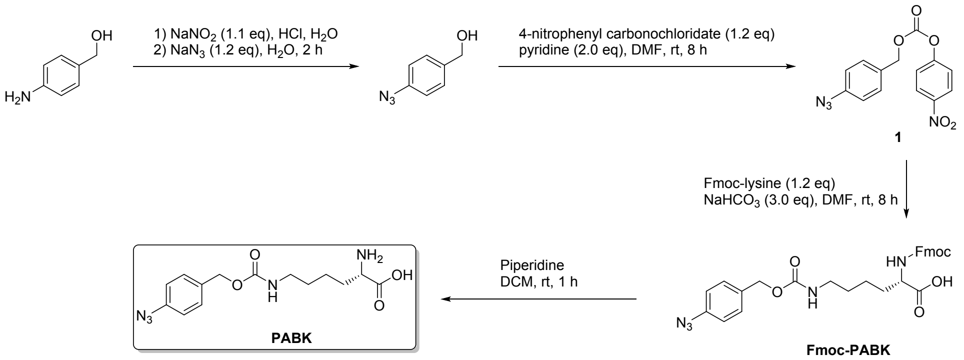

Supplementary Scheme 2. The synthesis route of PABK.

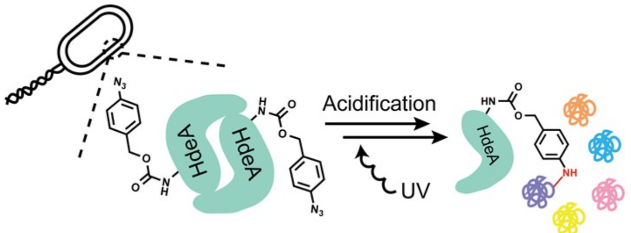

Supplementary Scheme 3. The model for HdeA photo-affinity capture in the periplasm of *E. coli* after acidification (pH = 2) using PABK as the photocrosslinking probe. Chaperone HdeA exists as a dimer form in the periplasm of *E. coli* cells at neutral pH. At low pH (< 3), HdeA homodimer dissociates to give the monomer for client proteins' protection. After UV-irradiation, HdeA bearing PABK can crosslink other proteins.

Supplementary Figures

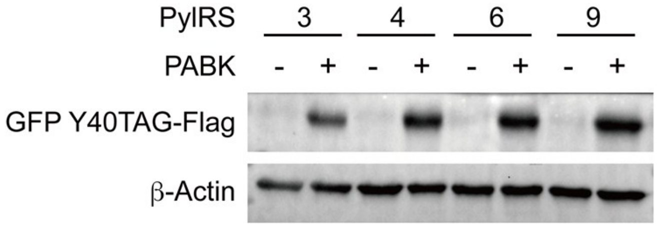

Supplementary Figure 1. Immunoblotting analysis of PABK incorporation into GFP-Y40TAG in the presence of different PylRS-tRNA pairs. These four PylRS-tRNA pairs are chosen from reported PylRS-tRNA pairs, which own different mutations listed on Table S1.

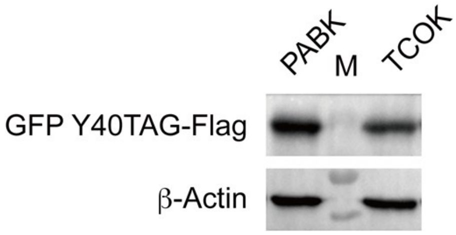

Supplementary Figure 2. Immunoblotting analysis confirming that the expression level of GFP carrying PABK is higher than GFP with TCOK. PABK was recognized by PylRS-9. TCOK was recognized by PylRS-3 as previously reported. After transient transfection and cell culture for 24 h in the presence of 1 mM PABK, nearly 16 μg GFP-Y40PABK(Flag) can be purified from 10^8 HEK293T cells.[6] M: protein marker ladder.

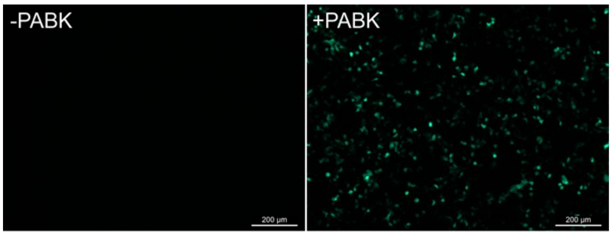

Supplementary Figure 3. Incorporation of PABK into GFP-Y40TAG in HEK293T cells. The transfected HEK293T cells were incubated at 37 °C with (+) and without (-) 1 mM PABK in the cell culture media for 24 h before being visualized by a fluorescence microscope. Scale bar: 200 μm.

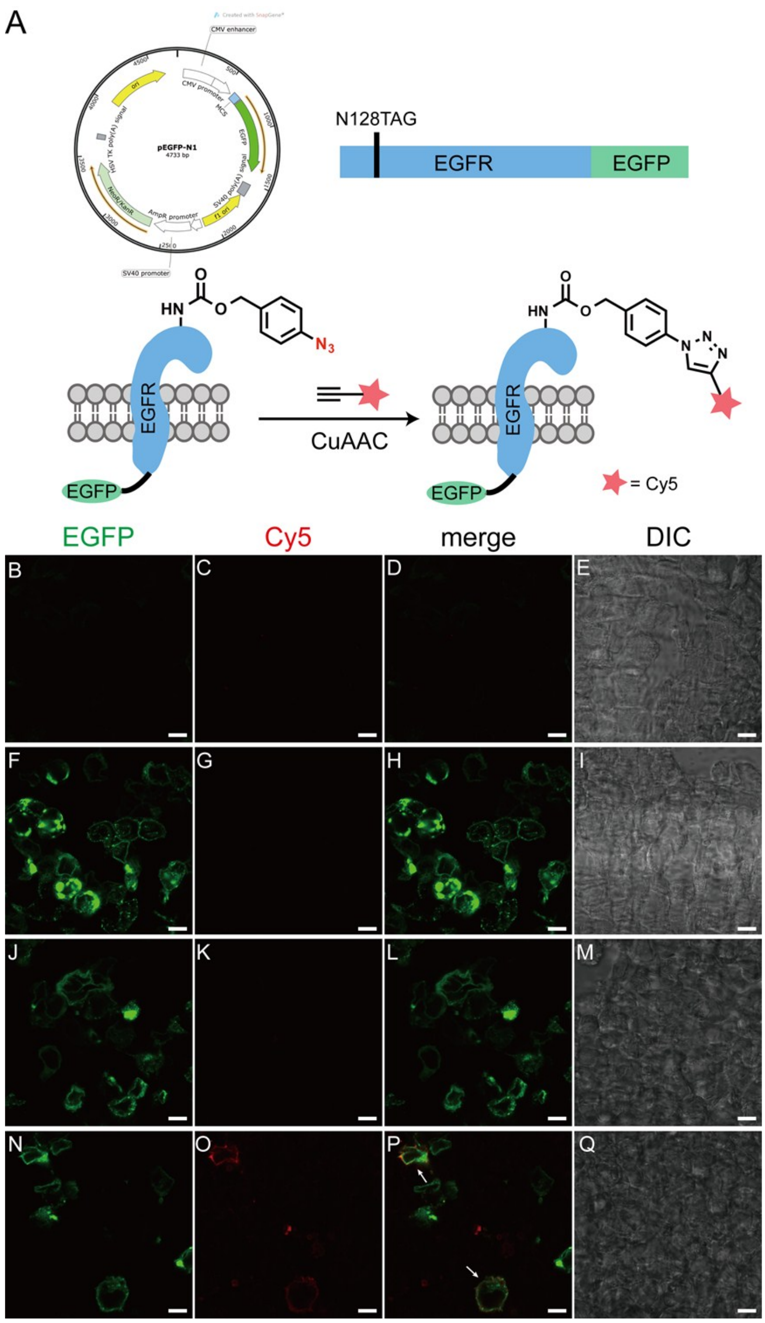

Supplementary Figure 4. Bioorthogonal labeling of EGFR-N128PABK-EGFP. (A) Schematic representation for EGFR-N128PABK-EGFP labeling at the cell membrane by a BTTAA-assisted CuAAC reaction. Upper left: the vector map of pEGFP-N1 (created with SnapGene®). Upper right: the schematic representation for EGFR construct. EGFR sequence was incorporated into MCS (multiple cloning site) followed by an EGFP (Enhanced Green Fluorescence Protein) sequence, which can express an EGFR with a C-terminal EGFP tag. The N128 site was mutated to the amber condon for UAA incorporation. (B)-(E) represent EGFR-N128TAG-EGFP without the addition of PABK, Cu(I)-BTTAA and alkyne-Cy5. (F)-(I) represent EGFR-N128TAG-EGFP expression with 1mM PABK in the absence of Cu(I)-BTTAA and alkyne-Cy5. (J)-(M) represent EGFR-N128PABK-EGFP labeling with alkyne-Cy5 without Cu(I)-BTTAA catalysis. (N)-(Q) represent EGFR-N128PABK-EGFP labeling with alkyne-Cy5 assisted by Cu(I)-BTTAA. The first column: EGFP channel; the second column: Cy5 channel; the third column: merge; the last

column: DIC. Scale bar: 10 μm. Cell line: HEK293T cells.

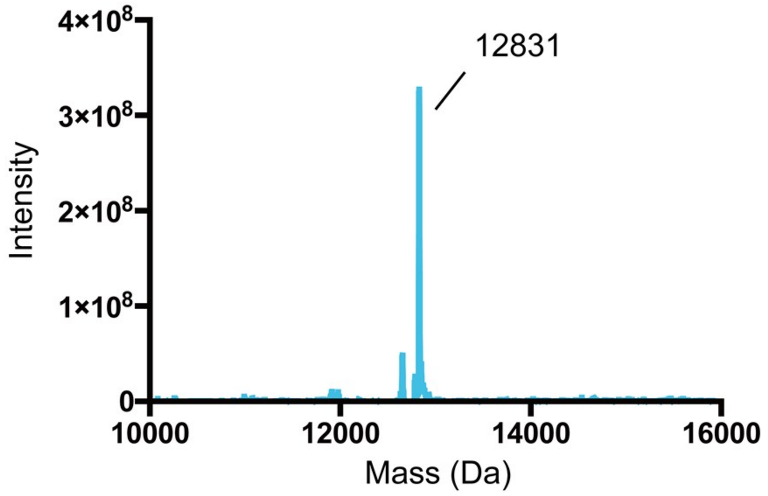

Supplementary Figure 5. ESI-MS data for PABK incorporation in HdeA V58 site. For HdeA-V58PABK-His: calculated value is 12835 Da, found 12831 Da. This data confirmed that PABK has been site-specifically incorporated into HdeA with high fidelity in *E. coli* cells.

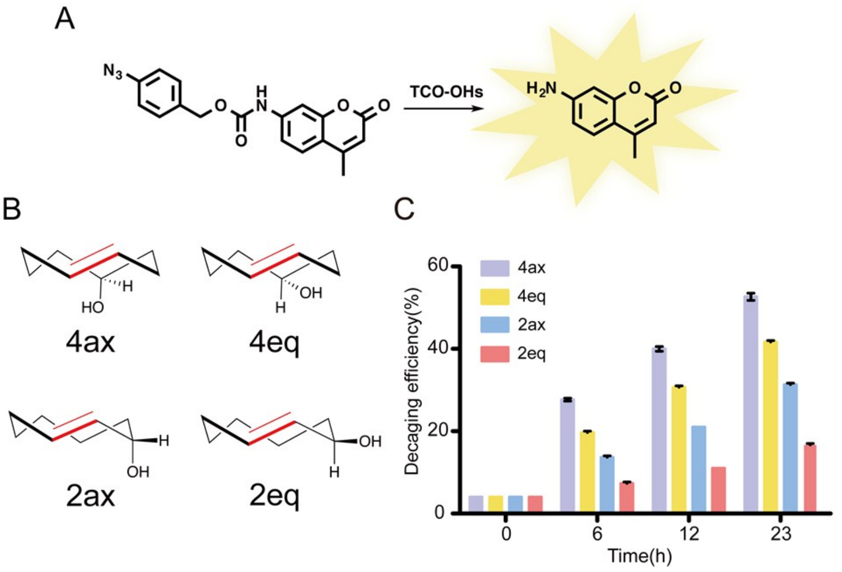

Supplementary Figure 6. Activator screening for TCO-OHs-mediated decaging chemistry. (A) Coumarin-based fluorogenic reporter for the TCO-PAB decaging reaction. (B) Structures of four TCO-OHs (named 4ax, 4eq, 2ax and 2eq respectively) screened in this study. (C) Coumarin Fluorescence assay displayed distinct decaging efficiencies of the TCO-derivatives shown in (B). Error bars represent ±1 SEM.

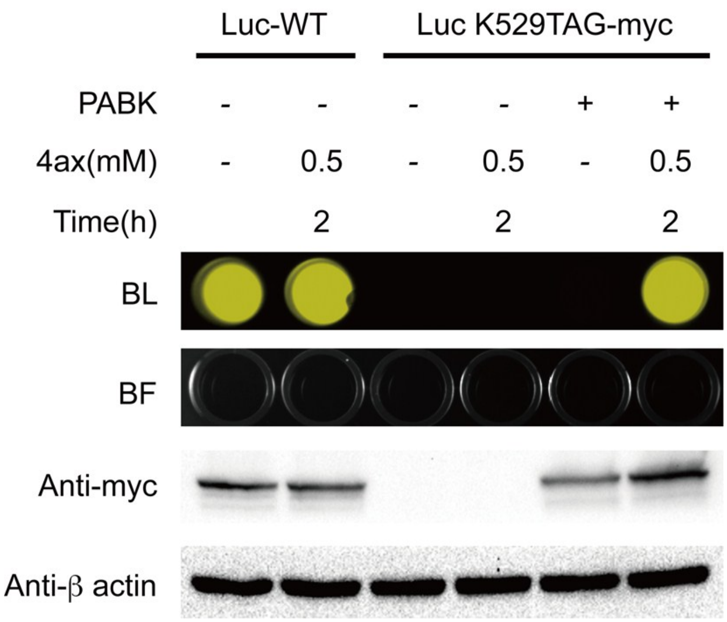

Supplementary Figure 7. Firefly luciferase-K529PABK(myc) can be activated by 4ax in mammalian cells. HEK293T cells expressing fLuc with or without 1mM PABK were treated with 4ax. Bioluminescence was analyzed by the chemiluminescent channel of ChemiDoc (BL) with bright field (BF) images taken as a control. The immunoblotting results aimed to show the expression level of fLuc-PABK and fLuc-wt (anti-myc), as well as the equal loading control (anti-β actin).

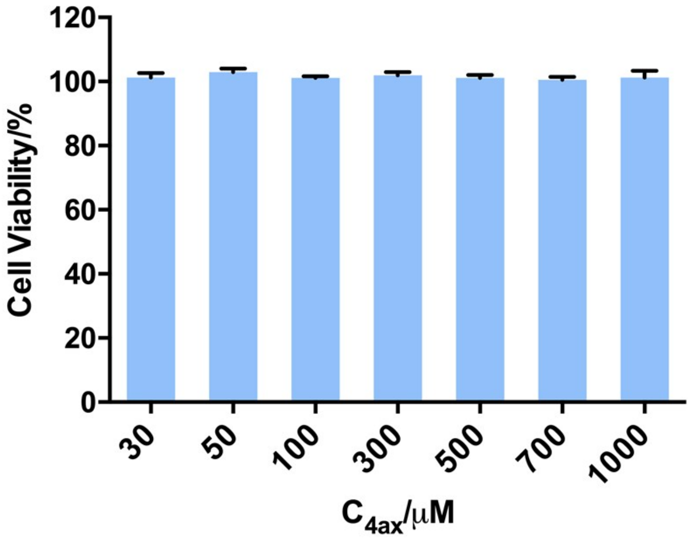

Supplementary Figure 8. MTS assay of 4ax. HEK293T cells were treated with 4ax at different concentrations for 3 h before being analyzed by MTS assay. Error bars represent ±1 SEM from three independent experiments.

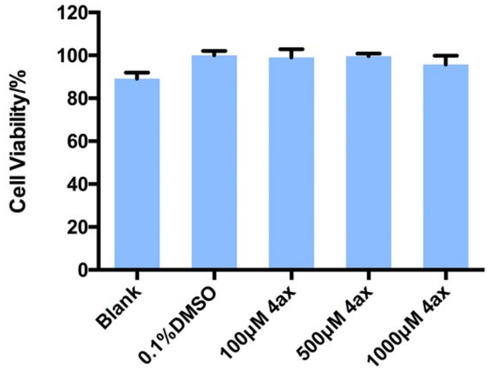

Supplementary Figure 9. MTS assay of 4ax for 72h. HEK293T cells were treated with 4ax at different concentrations for 72 h before being analyzed by MTS assay. Error bars represent ±1 SEM from three independent experiments.

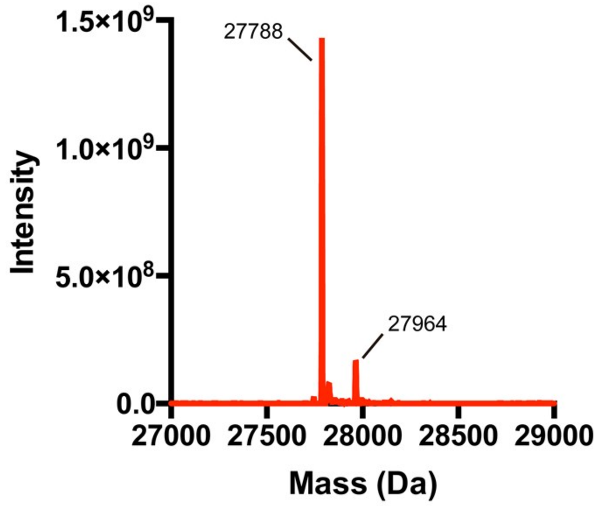

Supplementary Figure 10. ESI-MS data for decaging reaction between PABK incorporated GFP and 4ax in cell lysate. HEK293T cells expressing GFP-Y40PABK(Flag) were lysed via sonication. The cell lysate reacted with 4ax (500 μM final concentration) at 37 °C for 4 h. For GFP-Y40PABK(Flag), calculated value: 27967 Da, found 27964 Da. For GFP-Y40K(Flag), calculated value: 27792 Da, found 27788 Da. The decaging percentage is about 90%.

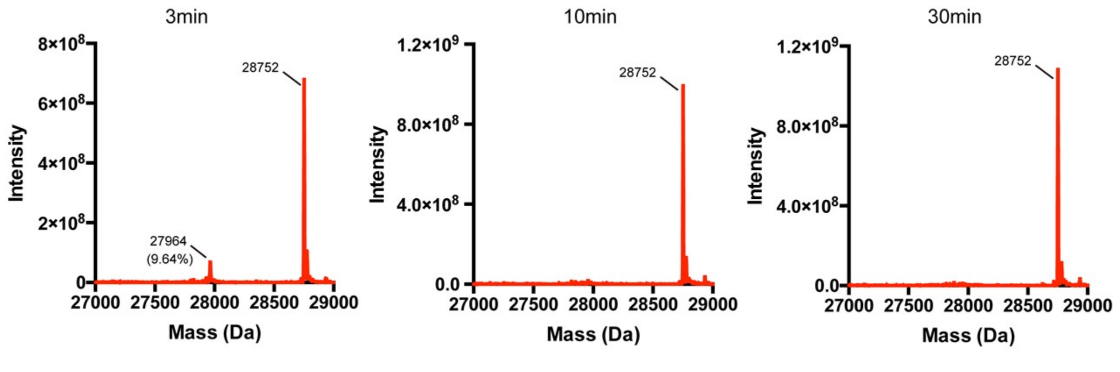

Supplementary Figure 11. ESI-MS data for CuAAC reaction between PABK incorporated GFP and Alkyne-Cy5 in vitro. The purified GFP-Y40PABK(Flag) incubated with Alkyne-Cy5

for different time. The reactions were quenched by BCS and the labeling efficiency was determined by LC-MS. After 3 min, only 9.64% GFP-Y40PABK(Flag) remained and more than 90% proteins have been converted to the addition product (GFP-Y40PABK-Cy5). After 30 min, only the addition product can be observed, which demonstrated that the bio-conjugation reaction on PABK cannot lead to any cleavage process. For GFP-Y40PABK(Flag), calculated value: 27967 Da, found 27964 Da. For GFP-Y40PABK-Cy5, calculated value: 28756 Da, found 28752 Da.

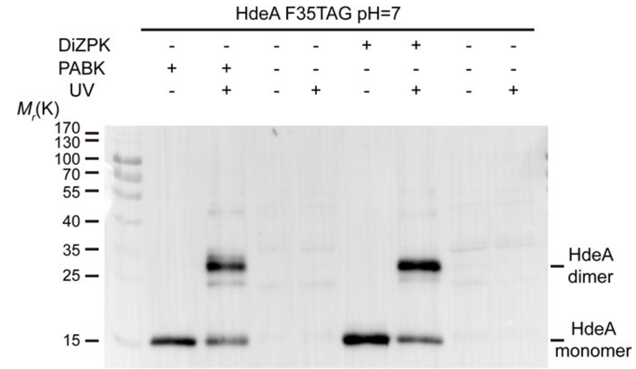

Supplementary Figure 12. Immunoblotting analysis of the photocrosslinking efficiency with PABK or DiZPK incorporating in model HdeA protein. *E. coli* cells expressing HdeA-F35PABK or HdeA-F35DiZPK were irradiated then photocrosslinked at physiology condition (pH=7). Clean HdeA dimerization at pH 7 can be observed. [7]

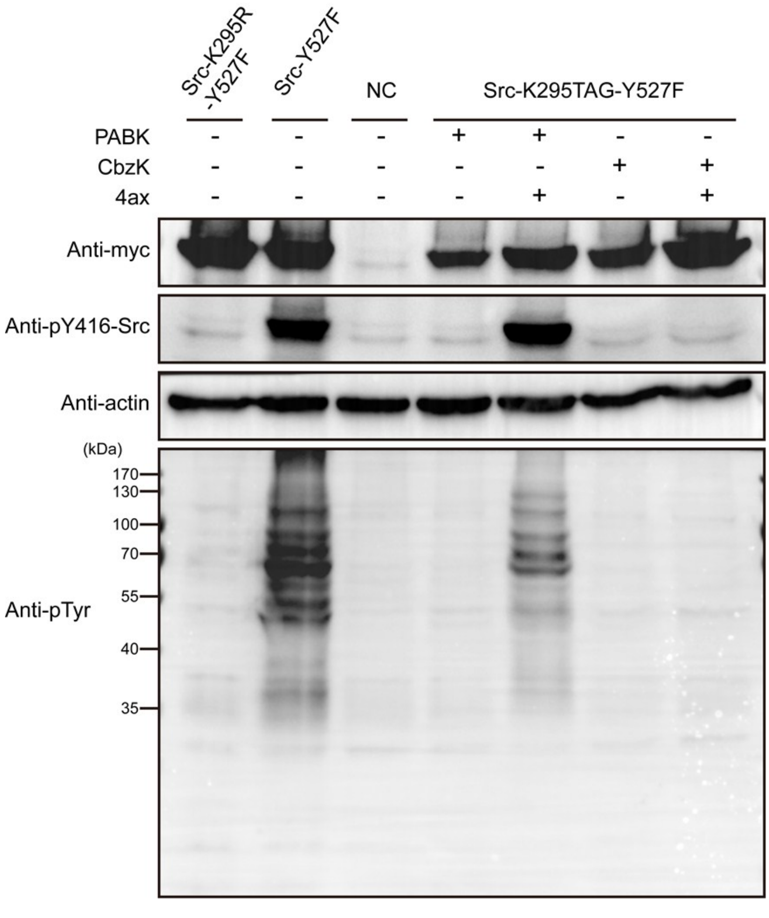

Supplementary Figure 13. Src-K295PABK-Y527F(myc) can be activated by 4ax in HEK293T cells. The activity of Src can be suppressed via replacing the key site K295 with PABK or CbzK (N6-Carbobenzyloxy-L-lysine). Only the Src-K295PABK-Y527F can be reactivated after incubating with 4ax (500 μM) for 3 h, which up-regulated auto-phosphorylation level and downstream phosphorylation level (lane 5). Src-Y527F is a constitutive active form of Src and Src-K295R-Y527F is an inactive form of Src. [8,9]

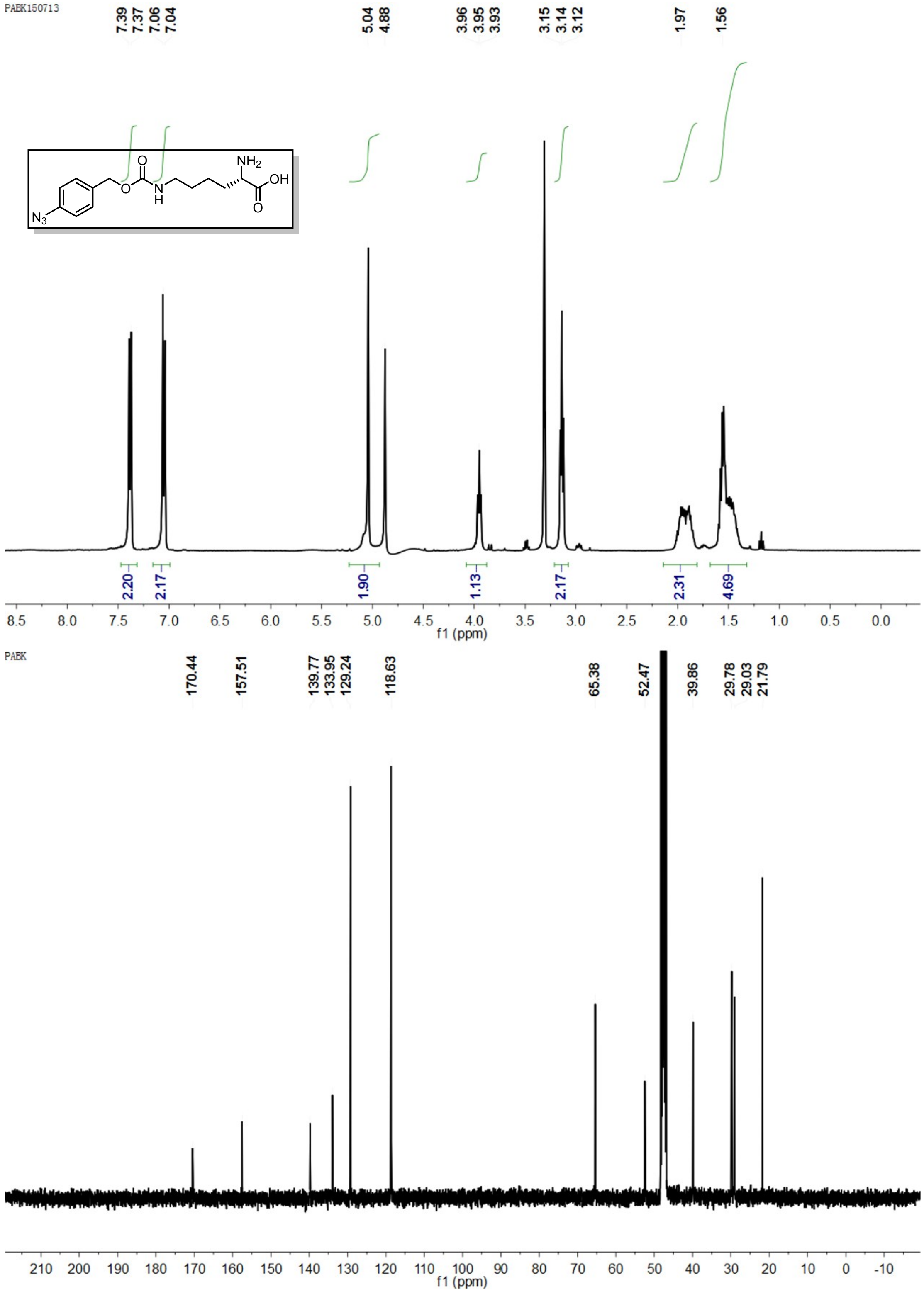

Supplementary Figure 14. NMR spectra of PABK.

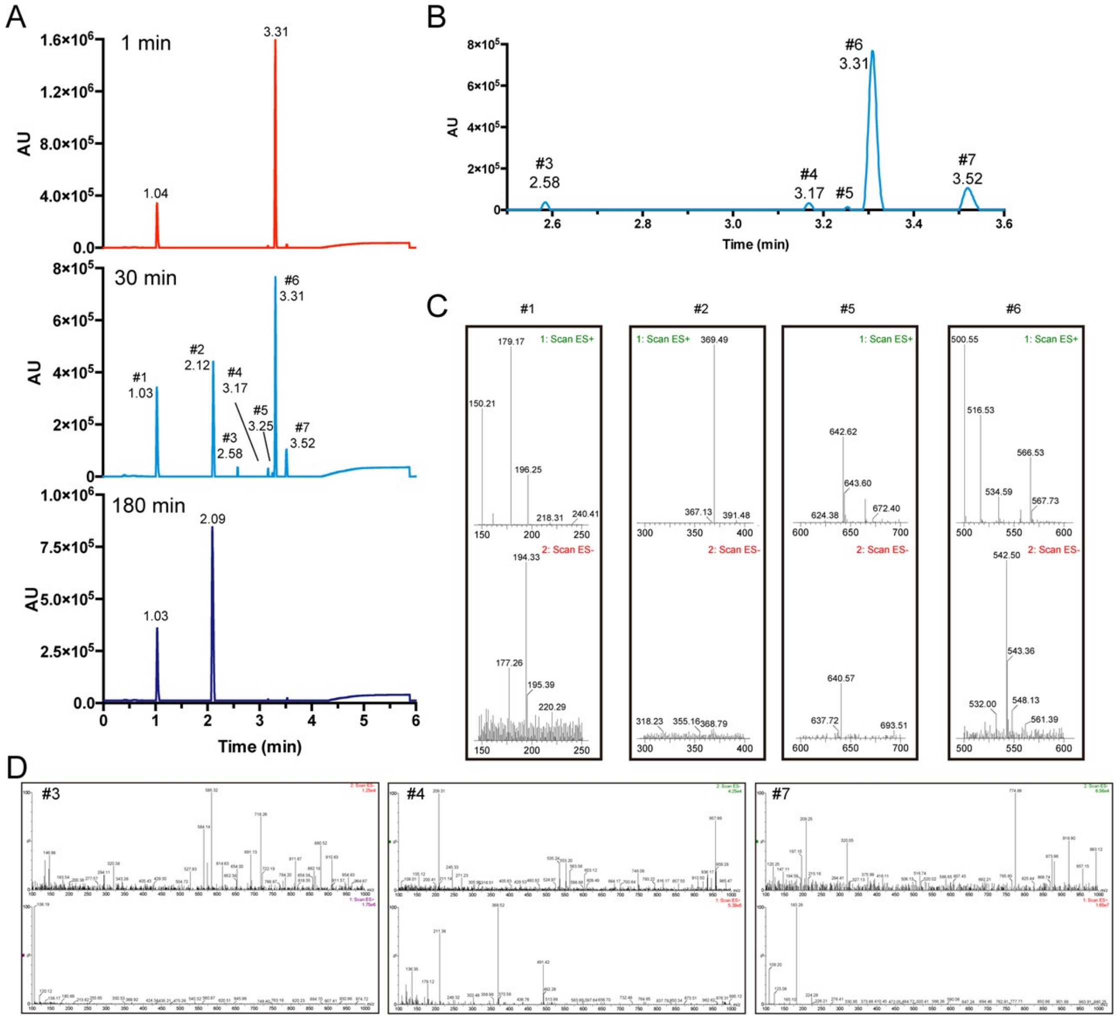

Supplementary Figure 15. Detailed LC-MS spectra of reactions between Fmoc-PABK and 4ax. (A) Full LC spectrum of reaction between Fmoc-PABK and 4ax at 1 min, 30 min and 180 min. Peaks are labeled with corresponding LC time. Part of the LC spectrum were enlarged and compiled in Figure 4B. (B) LC spectrum of reaction between Fmoc-PABK and 4ax (rescaled for better resolution) at 30 min. (C) MS spectra of identified peaks from above LC spectrum (top: positive ion mode; bottom: negative ion mode). #1: internal standard compound (N-methyl tyrosine, MW = 195.1); #2: Fmoc-lysine (decaged product, MW = 368.2); #5: Intermediate (MW = 641.3); #6: Fmoc-PABK (starting material, MW = 543.2). (D) MS spectra of the tiny peaks (#3, #4 and #7), which are unable to be identified (top: negative ion mode; bottom: positive ion mode).

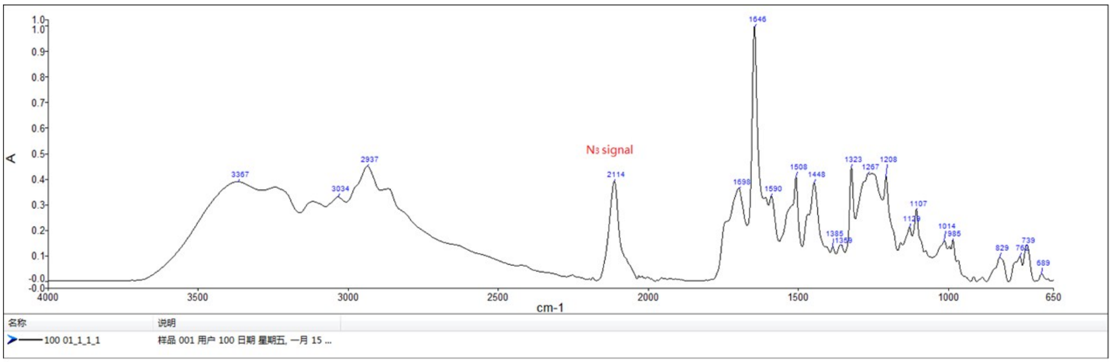

Supplementary Figure 16. Infrared spectrum of PABK.

References

1 R. van Brakel, R. C. M. Vulders, R. J. Bokdam, H. Grull, M. S. Robillard, *Bioconjugate Chem.* 2008, **19**, 714-718.

2 S. S. Matikonda, D. L. Orsi, V. Staudacher, I. A. Jenkins, F. Fiedler, J. Chen and A. B. Gamble, *Chem. Sci.,* 2015, **6***,* 1212-1218.

3 M. Royzen, G. P. A. Yap and J. M. Fox, *J. Am. Chem. Soc.,* 2008, **130***,* 3760-3761.

4 M. Zhang, S. Lin, X. Song, J. Liu, Y. Fu, X. Ge, X. Fu, Z. Chang, P. R. Chen, *Nat. Chem. Biol.* 2011, **7**, 671-677.

5 Y. Yang, S. Lin, W. Lin, P. R. Chen, *Chembiochem* 2014, **15,** 1738-1743.

6 J. Li, S. Jia, P. R. Chen, *Nat. Chem. Biol.* 2014, **10**, 1003-1005.

7 S. Lin, D. He, T. Long, S. Zhang, R. Meng, P. R. Chen, *J. Am. Chem. Soc.*, 2014, **136**, 11860-11863.

8 Y. Qiao, H. Molina, A. Pandey, J. Zhang, P. A. Cole, *Science* 2006, **311**, 1293-1297.

9 G. Zhang, J. Li, R. Xie, X. Fan, Y. Liu, S. Zheng, Y. Ge and P. R. Chen, *ACS Cent. Sci.*, 2016, **2**, 325-331.
